# Mobile electromagnetic levitator for time-resolved *in situ* X-ray diffraction studies of high-temperature phase transformations

**DOI:** 10.1107/S1600577526003541

**Published:** 2026-05-06

**Authors:** Olga Shuleshova, Ivan Kaban, Steffen Ziller, Uwe Reinhold, Ann-Christin Dippel, Olof Gutowski, Martin von Zimmermann

**Affiliations:** a Leibniz Institute for Solid State and Materials Research Dresden Helmholtzstr. 20 01069Dresden Germany; b Deutsches Elektronen-Synchrotron DESY Notkestr. 85 22603Hamburg Germany; Bhabha Atomic Research Centre, India

**Keywords:** electromagnetic levitation, synchrotron X-rays, time-resolved diffraction, phase transformations, metastable solidification

## Abstract

*In situ* observations of high-temperature transformations, not compromised by reactivity with a crucible, have recently become accessible by integrating containerless processing with the time-resolved diffraction of synchrotron X-rays. The presented electromagnetic levitator (EML) has been designed for mobility and ease of operation during beamline experiments while enabling stable processing of a wide range of materials. Developed to work with area detectors, the facility’s capabilities are demonstrated by measurements of the structural changes during melting and solidification of pure Fe and Fe–Co samples. The direct observations of the metastable phase formation in the Fe–Co system, accessible through containerless processing, have been accomplished for the first time due to the improved acquisition rate of modern area detectors. The diffraction patterns and associated artifacts characteristic of crystal structure measurements during levitation are examined. Moreover, an analytical description of the measured instrumental resolution of the diffraction setup is presented along with a survey of the corrections and associated measurements necessary for an accurate total scattering analysis of the disordered states, such as liquids or glasses. This provides a base for the conceptual planning of diffraction experiments suitable for various levitation methods, including electrostatic, aerodynamic and acoustic levitation.

## Abstract 2

A mobile electromagnetic levitator combined with time-resolved synchrotron X-ray diffraction enables *in situ* studies of high-temperature phase transformations without container-induced contamination and reveals the first direct observation of metastable phase formation in undercooled Fe–Co alloys.

## 1. Introduction

Advancements in adapting containerless processing techniques for scattering experiments have enabled unobscured observations of structure evolution in high-temperature, reactive materials (Shuleshova *et al.*, 2012 ▸; Herlach & Matson, 2012 ▸; Kelton & Gangopadhyay, 2005 ▸). Measurements have been conducted at synchrotron and neutron sources with levitation facilities operating on electromagnetic (Notthoff *et al.*, 2000 ▸), electrostatic (Mauro & Kelton, 2011 ▸; Kordel *et al.*, 2011 ▸; Mauro *et al.*, 2016 ▸) and aerodynamic (Mizuno *et al.*, 2007 ▸) principles. Unique access to metastable liquid and solid phases, achieved by eliminating interactions with container walls, has driven the main part of the research. As neutron scattering experiments are generally restricted due to long acquisition times to measurements at constant temperatures, structural evolution during temperature changes is typically accessed using synchrotron X-rays. Photons of sufficiently high energy and flux can penetrate bulk levitating samples in transmission geometry, allowing real-time observation of successive phase transformations. The first electromagnetic levitator (EML) designed for synchrotron diffraction employed a white, polychromatic X-ray beam and an energy-dispersive detector (Notthoff *et al.*, 2000 ▸). A minimum acquisition time of 0.5 s (2 Hz) was required to ensure sufficient counting statistics for the diffracted intensity. A few years later, the electrostatic levitator (ESL) developed for experiments with a monochromatic beam took advantage of evolving area detectors, enabling acquisition rates up to 40 Hz (Gangopadhyay *et al.*, 2005 ▸).

Time-resolved experiments allowed *in situ* observation of solidification pathway changes with increasing melt undercooling, capturing transient metastable phase formation (Notthoff *et al.*, 2000 ▸; Shuleshova *et al.*, 2011 ▸). Supported by neutron diffraction experiments, continuous X-ray diffraction (XRD) measurements revealed subtle thermal changes in the topological and chemical short-range order of metallic alloys below the liquidus temperature, suggesting their connection to glass-forming ability (Wei *et al.*, 2013 ▸; Mauro *et al.*, 2013 ▸; Kaban *et al.*, 2013 ▸; Gangopadhyay *et al.*, 2014 ▸; Kaban *et al.*, 2014 ▸). Enhanced diffusion at high temperatures was shown to allow the unambiguous identification of solid–liquid equilibrium phases, regardless of system complexity (Shuleshova *et al.*, 2010 ▸; Gegner *et al.*, 2013 ▸). The temperature evolution of lattice constants determined during continuous heating has been successfully used to trace phase boundaries and capture liquid–liquid miscibility gaps in binary alloy systems (Mattern *et al.*, 2012 ▸; Mattern *et al.*, 2013 ▸).

Discussions on instrumental resolution and the interpretation of two-dimensional diffraction data from levitated spherical samples remain limited within the extensive body of research, despite the need for detailed consideration of this subject. For example, the implicit improvement in spatial resolution by reducing the beam size to a fraction of the sample, combined with limitations in sample alignment along the scattering axis, leads to an azimuthal asymmetry of the diffracted intensity due to absorption gradients. Crucial for accurate determination of the structure of liquids and glasses, this issue has been successfully addressed elsewhere (Zeidler, 2012 ▸; Bendert *et al.*, 2013*a* ▸; Bendert *et al.*, 2013*b* ▸). However, for the Bragg diffraction from the solid constituents, a systematic analysis of the effects of the experimental setup and the particular behavior of the levitating sample is still lacking.

The development of the third-generation synchrotron sources, such as PETRA III at DESY (Deutsches Elektronen-Synchrotron), and flat-panel detectors with acquisition rates enhanced by an order of magnitude opened up new possibilities for significant improvement in time resolution without compromising the quality of measured data. Presented here, a mobile electromagnetic levitation facility designed at Leibniz IFW Dresden for time-resolved *in situ*X-ray diffraction measurements with area detectors makes use of these technological advancements.

Compared with other levitation techniques, electromagnetic processing ensures stable sample positioning across a wide range of materials, including those containing volatile elements. Wide diffraction angles, 2θ, enabled by EML design, along with the short wavelength, λ, of the high-energy photons (around 100 keV) provide optimal reciprocal space coverage, as the wavevector is determined by *Q* = 4πsinθ/λ. The discussion on the boundaries of achievable resolution in *Q*-space is driven by dedicated measurements and analytical analysis of parameters determining instrumental resolution. This assessment offers a practical tool for optimizing diffraction setups with levitation sample environments to meet specific experimental goals.

The characteristic evolution of diffraction patterns observed during levitation upon melting and solidification are illustrated with *in situ* diffraction experiments on pure iron. A case study on the metastable solidification of Fe–Co alloys demonstrates the unique capabilities of the levitation facility, which is equipped for time-resolved structural studies with a fast area detector, to capture dynamic events, such as rapid phase transformations on the millisecond timescale. Complemented by a detailed analysis of potential artifacts, this work provides the first comprehensive guide for interpreting time-resolved diffraction results during phase transformations in freely suspended spherical samples.

## 2. Apparatus

### 2.1. Mobile electromagnetic levitator

**Figure 1:**
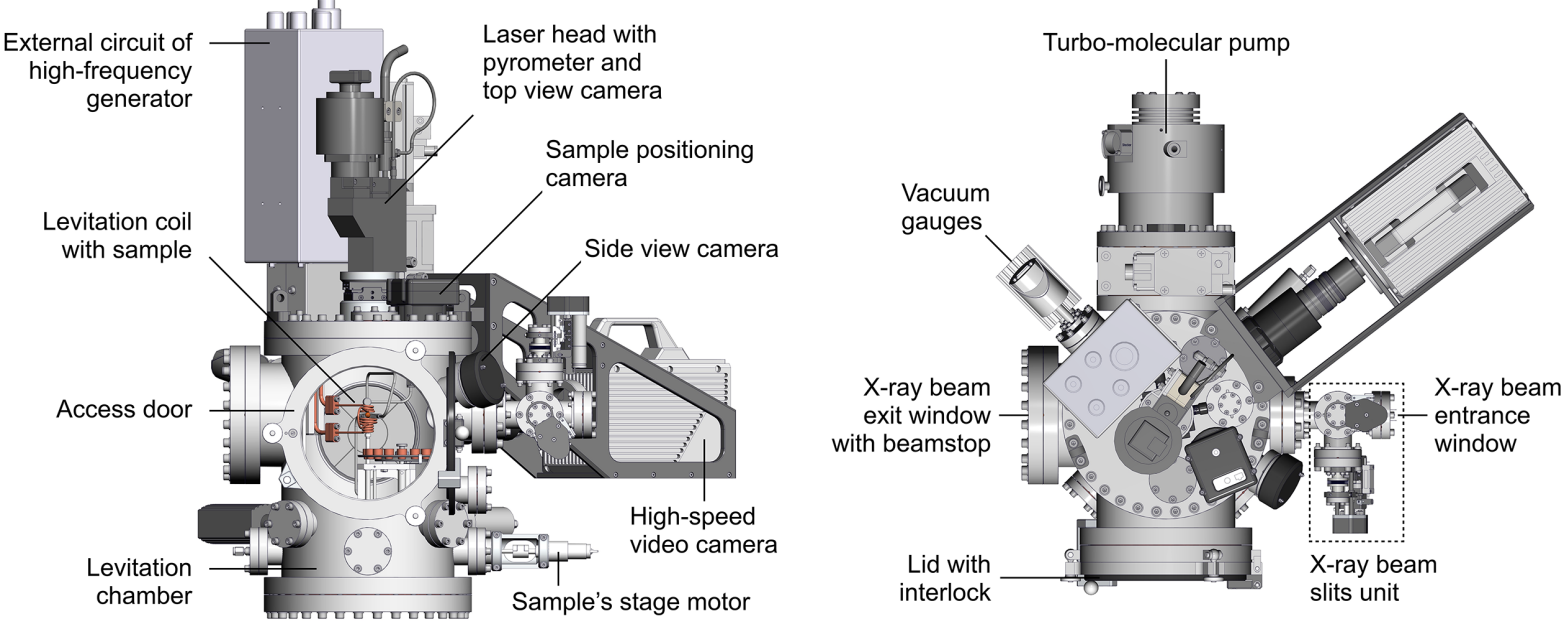
3D rendering of the mobile EML chamber with key components shown in the front (left) and top (right) view.

A mobile electromagnetic levitator dedicated to diffraction studies with synchrotron X-rays has been developed and constructed at IFW Dresden. The facility features four units: a levitation chamber with the equipment shown in Fig. 1 ▸, a supporting rack with the vacuum and gas supplies, an electronic rack, and an experiment control and display unit.

**Figure 2:**
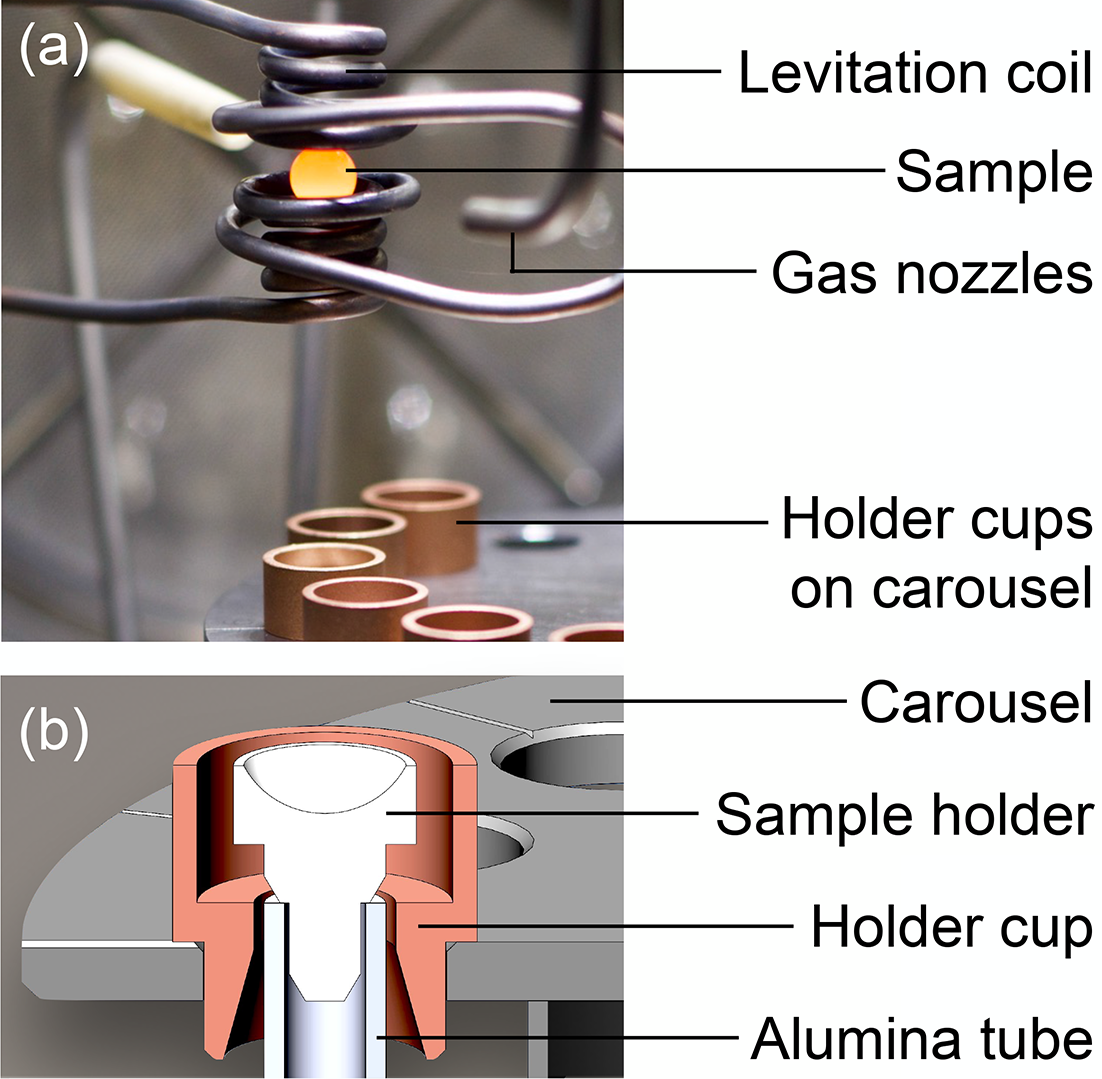
Close-up view of the sample levitating within the induction coil (*a*); a cross-section view of the sample holder assembly (*b*).

Levitation experiments are carried out in a high-vacuum cylindrical chamber of 250 mm diameter (stainless steel, by VACOM), which is fitted with 16 side-welded and 6 top-welded ConFlat flanges (DN40, unless specified otherwise). The samples rest on specially designed alumina holders, placed inside copper cups and loaded through a glass access door of 200 mm diameter onto a carousel that accommodates up to 16 samples. The cross section of the sample holder assembly is detailed in Fig. 2 ▸(*b*). The carousel is rotated by an electric gear (Serie PG 30/1S, MATTKE AG) via the feedthrough in the lower part of the chamber. The second gear enables vertical movement of the 6 mm-diameter alumina tube used to lift the ceramic holder with the sample into the processing position within the levitation coil.

Once the samples are loaded, the chamber can be evacuated up to 10^−8^ mbar using a vacuum system through the DN150 flange situated at the rear of the chamber. The pre-vacuum pump (SCROLLVAC SC 30D, Oerlikon Leybold Vacuum) is mounted within the supporting rack on a 60 mm × 750 mm × 750 mm granite block to minimize vibrations. The turbomolecular pump (TURBOVAC 361, Oerlikon Leybold Vacuum) is connected to the exhausting flange through the gate valve with a pneumatic actuator (DN160 CF EP, VA, 24V/DC, Oerlikon Leybold Vacuum). The vacuum level in the chamber is monitored by an ionization gauge (IONIVAC ITR 90, Oerlikon Leybold Vacuum) installed on a separate flange. The pressure around 10^−6^ mbar, suitable for most experiments, is typically achieved within 30 min.

The evacuated chamber is subsequently backfilled with noble gas of 99.9999% purity. It is possible to work with pure He, Ar or a He-5 vol.% H_2_ gas mixture. The supporting rack houses the gas cleaning and distribution system. After passing through an optional purification cartridge (Oxisorb, Messer Griesheim) the selected gas is directed by one of three diaphragm valves [HM20-4VKLC (M5), HAM-LET] via digital mass flow controllers (F-201AV-50K-ABD-33-V, Bronkhorst) to a common valve located just before the chamber. A ceramic diaphragm gauge (CERAVAC CTR 100, Oerlikon Leybold Vacuum) is used to accurately measure the chamber pressure for all gases. It is customary to work at pressure levels below 900 mbar to avoid the risk of potential contamination through the access door. A separate digital pressure controller (P-702CV, Bronkhorst) connected to the pre-vacuum pump maintains the constant gas pressure. In the event of a failure in the pressure control system, an additional mechanical overpressure valve (9SVM-14GA-VV-S, VACOM) activates at 1300 mbar.

The selected sample is processed within a high-frequency inductor positioned along the central axis of the chamber and powered through one of the top flanges by a 10 kW generator (TruHeat HF 5010, TRUMPF Hüttinger). The electromagnetic field at the nominal operating frequencies of 50–450 kHz is generated by an external circuit fixed to the top of the chamber, with the power supply situated within the electronic rack.

The inductor is a water-cooled cylindrical coil, hereafter referred to as the levitation coil [Fig. 2 ▸(*a*)], made from a 3 mm copper tube with a wall thickness of 0.5 mm. The coil is wound vertically with an inner diameter of 10 mm, maintaining a spacing of 1 to 2 mm between the turns. An alternating magnetic field generated by the coil induces eddy currents in the conductive sample, within the skin depth characteristic of the material. According to Lenz’s law, these currents produce a magnetic dipole moment that opposes the primary magnetic field. A resulting net diamagnetic repulsion force on a sample of up to 3 g mass is typically sufficient to overcome the gravitational force. To achieve stable sample levitation, the coil is designed with a few opposing turns at the top, creating a zero-force point for a sample of a given mass between the lower and upper sections, which are separated by a 10 mm gap.

Generated eddy currents simultaneously induce resistive heating of the sample due to ohmic losses. At a specific power level for a given material, the sample will reach its liquidus temperature, *T*_L_ (or melting point, *T*_m_, for pure elements), and melts. The liquid metal droplet levitates as long as its surface tension remains sufficiently high.

The temperature of the electromagnetically levitated sample is determined by the balance between the absorbed power and the heat transferred from the sample surface to the chamber environment. The differing dependence of power absorption and lifting force on the primary magnetic field allow for some independent variation in the sample temperature during levitation without significantly impacting its positioning (Herlach, 2014 ▸). Cooling can be further enhanced by directing gas streams onto the sample surface via two alumina nozzles on opposite sides. The same gas delivery and flow control system, utilized to establish the chamber atmosphere, is employed for sample cooling.

Alternative heating with a fiber-coupled diode laser (LM140, Mergenthaler) can be used for an inductively positioned sample. Operating at a wavelength of 938 nm, the laser delivers up to 140 W optical output at the end of the 600 µm fiber cable connected to the water-cooled power supply situated within the electronic rack. Protective measures, including interlocks, ensure that the facility complies with the safety standards required for a Class 1 laser.

The laser head (LH500-M) features a fiber-coupled pyrometer and a USB video camera, which are aligned coaxially with the laser beam path. It is positioned at the top of the chamber and directed through the collimation optics onto the sample. The video camera is used to adjust the desired size of the laser and pyrometer target spots on the sample surface.

The pyrometer continuously monitors the radiance temperature on the sample surface at a sampling rate of 100 Hz. It operates at wavelengths ranging from 1600 to 2100 nm in either single- or two-color mode with respective accuracy of 0.3 and 0.5% of measured value. The true temperature is calculated from the measured radiance temperature according to an established method based on Wien’s law (see the supporting information).

Sample positioning and processing within the coil are monitored laterally using a second USB video camera with an adjustable aperture.

To detect rapid thermal events characteristic of solidification from an undercooled state, the chamber is equipped with a high-speed video camera (775K/M1, PHOTRON Fastcam SA5). A 12-bit monochrome CMOS sensor with a pixel size of 20 µm captures the entire sample at a spatial resolution of 640 × 576 pixels, with an acquisition rate of 30000 frames per second (fps). The minimum exposure time required to achieve sufficient contrast usually restricts the acquisition rate to some 75000 fps.

One of the flanges is foreseen to accommodate an electrical feedthrough for a thermocouple to assist the pyrometer calibration and enable supplementary diffraction experiments at lower temperatures where levitation is not practical or desirable. In this case, the sample is processed on a modified alumina holder, which ensures contact with the thermocouple head. Heating is accomplished either by the levitation coil or the laser.

The programmable logic controller (SIMATIC IPC547C) controls the levitation system through the user interface. Each unit, such as valve activation, generator control, *etc.*, can be accessed individually in the manual operation mode. The automatic mode allows for the streamlining of the experiment with automated steps, including ‘Evacuation and gas filling’, ‘Experiment’ and ‘Chamber opening’.

### 2.2. Beamline experimental setup

**Figure 3:**
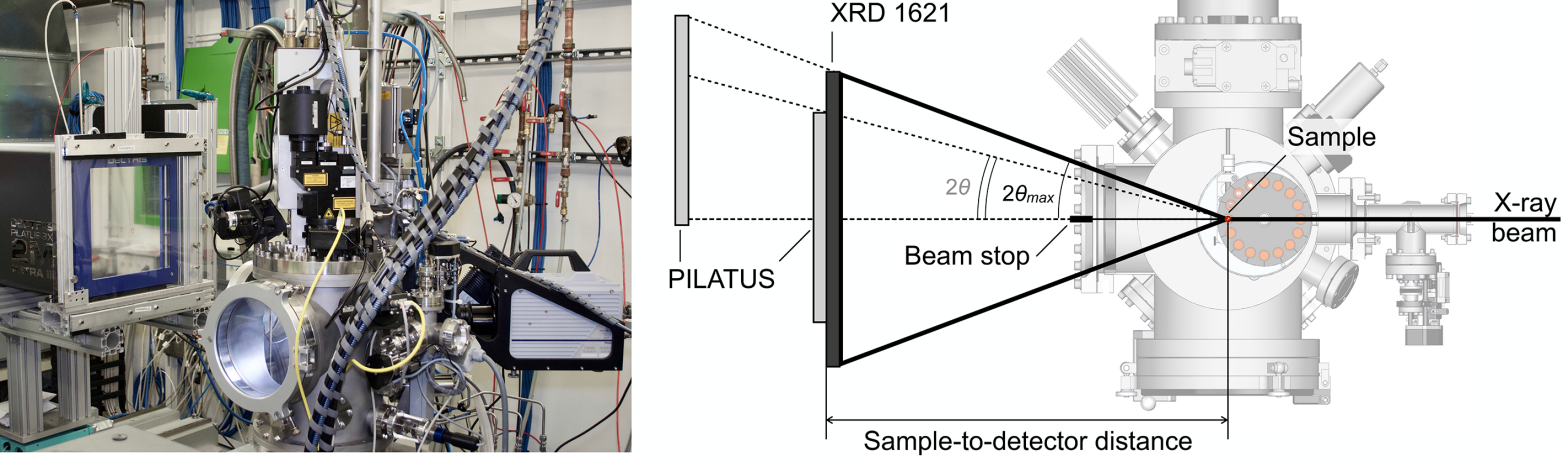
The mobile EML facility and PILATUS3 X CdTe 2M detector mounted at the P07 beamline at DESY (left). Symmetrical and off-center positions of the area detector used in transmission diffraction geometry for XRD measurements on levitated samples (right). Vertical dimensions of the sensitive areas of the PILATUS and XRD 1621 area detectors are depicted to scale.

The diffraction measurements using the mobile electromagnetic levitator have been conducted at the High-Energy Materials Science beamline P07 at DESY, Hamburg (Fig. 3 ▸). The synchrotron operates at 6 GeV particle energy, which, after passing through the undulator insertion device and Si double-crystal monochromator, delivers a flux of up to 7 × 10^11^ photons s^−1^ mm^−2^ at 100 keV with a bandwidth of 0.25%.

A photon beam of defined energy enters the experimental hutch via a beam shutter and goes through two slit systems that adjust the beam size. An iron attenuator can further reduce the intensity of the incident beam to protect the detector from damage.

For the beamline experiments, the levitation chamber is detached from the supporting rack and mounted on a motorized *xyz* table used to position the chamber in relation to the beam. Fig. 3 ▸ depicts the transmission geometry setup with the center of the levitating sample aligned with the beam axis. A set of motorized beam slits made of 1 mm tungsten are employed to cut off the scattering background after the beam passes through the entrance window of 5 mm-thick aluminium. The coil geometry enables unobstructed scattering of the incident beam by the sample. The diffracted beam passes through an exit window of diameter 150 mm made from 5 mm-thick AlMgSi1. Accessible *Q*-range in reciprocal space is limited by a 2θ_max_ ≃ 20°, which is determined by the size of the exit window and its distance from the sample. At the incident X-rays from 91 to 125 keV the range of the corresponding *Q*_max_ spans from 16 to 22 Å.

The residual primary beam and scattering background from the exit window are obstructed with a tantalum beam stop secured on the outer part of the exit window. A flat area-detector collects the scattered intensity transmitted through the exit window. A motorized support system that carries the detector is mounted on a common structural rail with the *xyz* table, enabling easy movement of the detector in relation to the sample position. The sample-to-detector distance (SDD) and the alignment of the detector to the incident beam (the scattering axis) are chosen according to the experimental objectives as detailed in Section 2.2.1.

To synchronize the thermal and structural information, the real-time data from the pyrometer and detector are recorded in a dedicated log file.

#### 2.2.1. Optimization of the experimental parameters

Sample sizes ranging from 4 to 7 mm diameter are selected based on their thermophysical properties (density, surface tension and electrical conductivity) to ensure stable processing in both solid and liquid states. To gain sufficient diffracted intensity after absorption in the sample and container, high-energy X-rays are required. Monochromatic radiation of 98.3 keV, corresponding to a wavelength of λ = 0.126186 Å, with a beam size of 0.5 mm × 0.5 mm is used for measurements throughout the present work (unless specified otherwise). With this, approximately 34% of intensity is transmitted through a spherical Fe sample of diameter 4 mm and 16% through a 7 mm sample.

Two flat area digital detectors deployed for high-energy X-ray diffraction and available at P07 beamline at DESY have been used in time-resolved electromagnetic levitation experiments: the XRD 1621 detector (PerkinElmer) and the PILATUS3 X CdTe 2M detector (DECTRIS). The main parameters of both detectors are compared in Table 1 ▸ (Förster *et al.*, 2019 ▸; PerkinElmer, 2006 ▸), with key performance issues discussed in Section 3.5. The diffraction patterns from both area detectors have been acquired and monitored using *QXRD* software (Jennings, 2015 ▸).

The geometric parameters of the diffraction setup required for detector calibration are derived from measurements of a standard material (calibrant). Current experiments have utilized the LaB_6_ and CeO_2_ powders, placed in a capillary along the central axis of the levitation coil, and exposed to the selected wavelength and beam size. The Debye–Scherrer cones scattered from the calibrant intersect the detector plane, producing a pattern of elliptical rings. Fitted by the calibration tool of the Python fast azimuthal integration library, *pyFAI* (Ashiotis *et al.*, 2015 ▸; Kieffer *et al.*, 2020 ▸), the geometry of the diffraction rings is used to determine the beam center and the point of normal incidence of the sample position on the detector plane (coincide when the detector is perfectly orthogonal to the scattering axis). The SDD is then refined based on the known diffraction angles of the calibrant at the operating wavelength. These calibrations allow the conversion of distances measured on the detector with the origin at the beam center into diffraction angles 2θ. Used *pyFAI* software transforms the two-dimensional diffraction patterns to polar coordinates (2θ, χ) and integrates scattered intensity along the azimuthal angle χ. The resulting one-dimensional patterns are recalculated from the 2θ angles into *Q*-space. Over-sampling of the *Q*-range bins by a factor of 3.5 has been used to obtain smoother intensity profiles.

*pyFAI* also implements intensity corrections due to the oblique incidence of the diffracted beam on the detector plane (solid angle corrections) and the polarization of the X-ray beam. A polarization factor of 0.99 was used for the current experiments. Other detector-specific corrections are addressed in Section 3.5.

**Figure 4:**
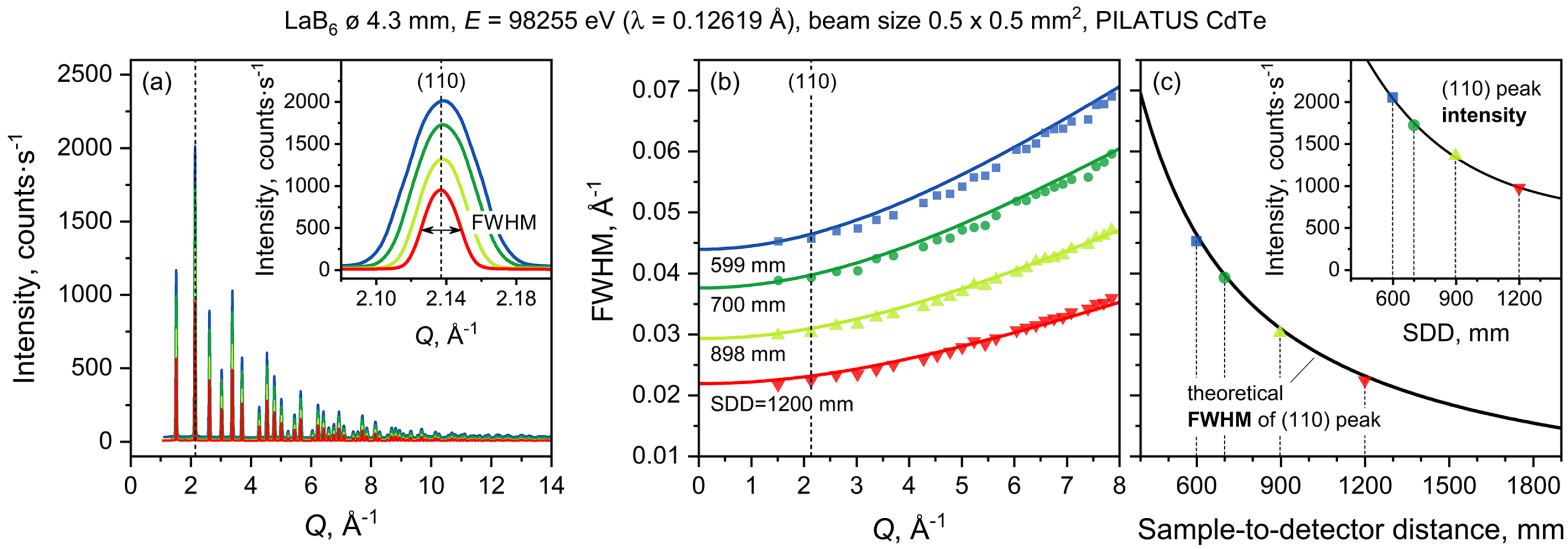
Instrumental resolution of the levitation diffraction setup: integrated diffraction patterns (*a*) and corresponding FWHMs (*b*) of LaB_6_ standard collected at different SDDs. Measured FWHM values (symbols) are compared with theoretical resolution (solid lines), as detailed in the supporting information. Dependence of the instrumental resolution on SDD (*c*), demonstrated with the FWHM of the main (110) diffraction peak of the LaB_6_ standard. Inserts illustrate the reduction of measured intensity due to air scattering as photons travel longer distances with increasing SDD.

**Figure 5:**
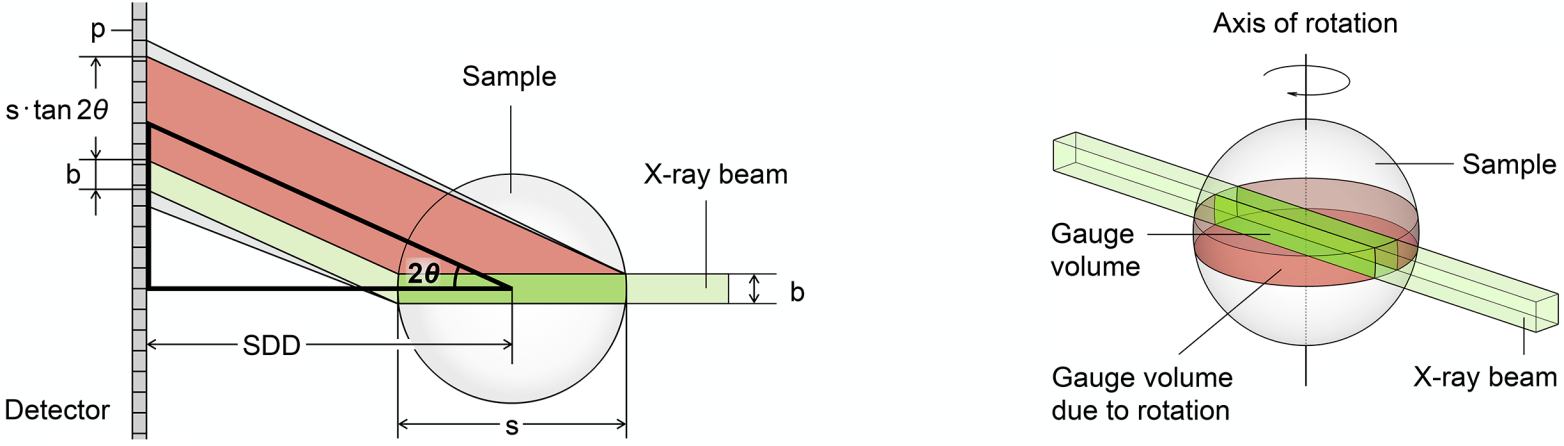
Simplified schematic of the geometrical origins of peak broadening at a given diffraction angle 2θ and sample-to-detector distance, arising from the finite sizes of the beam (*b*), sample (*s*), and detector pixels (*p*) (left). Schematic illustration of the gauge volume in diffraction experiments on a spherical sample under stationary and rotating conditions. Rotation of the sample leads to averaging over a larger effective volume, enhancing statistical sampling and reducing orientation-related artifacts (right).

Measurements of the standard material at various sample-to-detector distances have been used to determine the range of attainable instrumental resolution for a given experimental geometry. Fig. 4 ▸(*a*) shows integrated intensities for a series of measurements conducted at various SDDs using the LaB_6_ powder filled into a 4.3 mm-diameter capillary. The *Q*-dependence of the reflection widths (defined as the FWHM) indicates the instrumental resolution for a diffraction setup [Fig. 4 ▸(*b*)]. The experimentally measured values align well with the theoretical resolution function, calculated as a Gaussian convolution of the independent broadening contributions (see the supporting information for details). The agreement confirms that, for synchrotron X-ray energies around 100 keV, the effects of beam divergence and the intrinsic widths of the LaB_6_ reflections are negligible, and that the observed broadening is primarily determined by geometrical effects arising from the finite sample diameter, beam size, and detector pixel size depicted in Fig. 5 ▸. At a given sample-to-detector distance, the resolution at low *Q* is primarily limited by the beam size and the specifications of the area detector, *i.e.* pixel size and associated point spread function. At higher *Q* values (larger diffraction angles), it becomes progressively determined by the sample size. Increasing the sample-to-detector distance enhances angular resolution but reduces intensity due to air attenuation. As shown for the main (110) diffraction peak of the LaB_6_ standard in Fig. 4 ▸(*c*), the FWHM and intensity are inversely proportional to the SDD: doubling the SDD from about 600 to 1200 mm reduces both values by approximately half. The effect weakens with further increase of the sample-to-detector distance, providing only incremental improvements in resolution. At the same time, at larger SDDs, the finite detector size covers a smaller *Q*-range. Shifting the detector from its symmetrical position around the scattering axis can partly compensate for decreased reciprocal space coverage (see the off-center position in Fig. 3 ▸). However, this further restricts the azimuthal range for integration, which in turn lowers the average counting statistics.

## 3. Results and discussion

Essential aspects of time-resolved structural measurements, including diffraction patterns and artifacts characteristic of levitation experiments, are analyzed using elemental Fe. The binary Fe–Co system serves as a case study illustrating the capability of the area detector with a fast acquisition rate to capture rapid phase transformations in deeply undercooled melts produced by the levitation method. To maximize angular resolution, the measurements of pure Fe and Fe–Co alloys were conducted with a fast PILATUS detector at an SDD of 1500 mm. On the other hand, to capture full diffraction rings over a large *Q*-range, the XRD 1621 detector was set at a smaller SDD of 800 mm, and a higher photon energy of 121.3 keV was used.

Note that ferromagnetic materials must be heated above the Curie temperature (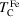 = 770°C) to be captured by the magnetic field of the levitation coil. The sample is preheated on the concave alumina holder, positioned at the central point between the upper and lower coil windings. Once the magnetic transformation is completed and the sample is stably positioned, the holder is lowered for further contactless processing.

The samples of ∼1 g mass have been produced from high-purity elemental Fe (99.995%) and Co (99.9%). The alloying was conducted in a high-purity Ar (99.999%) atmosphere using an arc-melting furnace, followed by homogenization and casting of the pre-alloyed buttons in a cold-crucible furnace. The composition of the as-cast rods of 8 mm diameter was confirmed using the inductively coupled plasma optical emission spectrometry (ICP-OES) method. Additionally, the composition homogeneity was verified using energy-dispersive X-ray spectroscopy (EDX) analysis. The mass of the samples cut from the elemental Fe stock or rods with nominal compositions of Fe_80_Co_20_ and Fe_70_Co_30_ was determined using a calibrated microbalance. The sample mass was thoroughly controlled before and after the levitation experiments to determine potential compositional changes due to material evaporation in the liquid state at high temperatures.

### 3.1. Pure Fe

**Figure 6:**
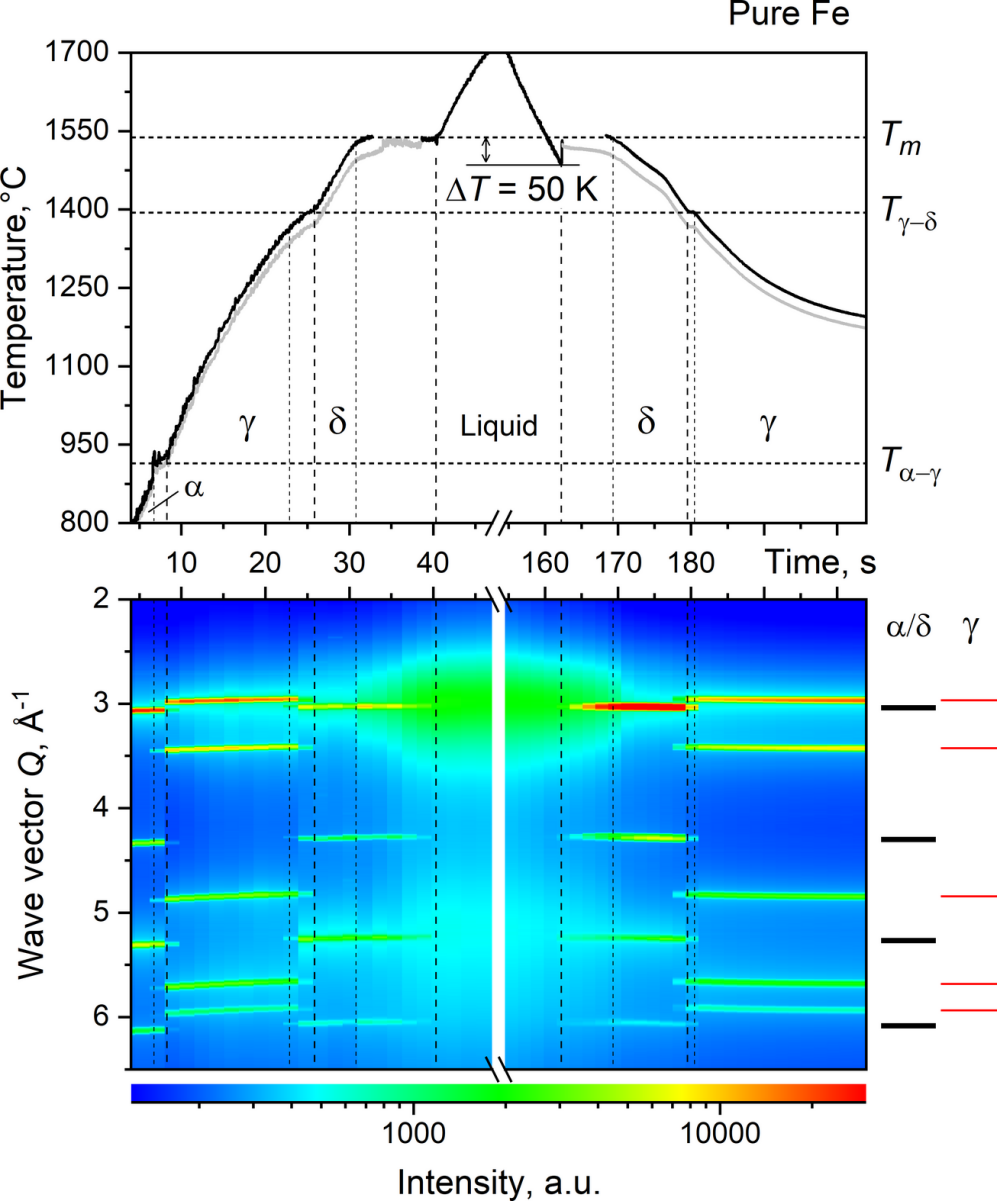
Structure evolution of elemental Fe processed with mobile EML: the intensity contour plot (bottom) of the integrated diffraction patterns measured at 1 Hz by the PILATUS detector, synchronized in time with the temperature profile recorded by the pyrometer (top). The true temperature (black line) is calibrated at *T*_γ–δ_ and *T*_m_ for the solid and liquid phases, respectively. The extrapolation of *T*_m_ calibration into the solid region (gray line) highlights the importance of phase-specific temperature calibrations for the solid sample.

Fig. 6 ▸ presents the continuous evolution of the elemental Fe structure measured by the PILATUS detector at a 1 Hz acquisition rate during heating and cooling in EML. The temperature is synchronized in time with integrated diffraction patterns. Upon heating, the crystalline α phase with a body-centered cubic (b.c.c.) structure first undergoes an allotropic transformation to γ of a face-centered cubic (f.c.c.) structure. However, at higher temperatures the b.c.c. structure of δ allotrope becomes stable again. Both transformations are accompanied by weak thermal responses at corresponding temperatures, *T*_α–γ_ and *T*_γ–δ_, while a more distinct temperature plateau is associated with the δ phase melting. Above the melting point, the diffraction patterns reveal only diffuse maxima characteristic of liquid. The liquid droplet is overheated above *T*_m_ to ensure the dissolution of the residual surface oxides. Cooling is achieved by reducing the coil current and activating the gas streams directed at the sample surface. As levitating droplets do not come into contact with the crucible, potential heterogeneous nucleation sites are dramatically reduced and the nucleation of the solid phase is often retarded. As shown in Fig. 6 ▸, the first thermal event during the cooling of the levitating Fe sample occurs below the melting point at nucleation temperature, *T*_N_. The spontaneous nucleation is followed by the rapid growth of the solid phase, with the driving force of transformation and the corresponding solid fraction being proportional to the undercooling attained in a given cycle, Δ*T* = *T*_m_ − *T*_N_ (Herlach *et al.*, 1993 ▸). After recalescence (a steep temperature rise) caused by the rapid release of latent heat, the solidification of residual liquid proceeds under near-equilibrium conditions at *T*_m_. The Bragg reflections of the δ phase are observed during solidification and further cooling. The emergence of the γ reflections aligns with the weak plateau at *T*_γ–δ_, indicating δ to γ solid-state transformation. Note that the increase in cooling rate between *T*_m_ and *T*_γ–δ_ results from the heightened flow of cooling gas and does not involve any structural changes. Before the Curie temperature is reached, the coil power is turned off, and the sample drops onto a holder. The thermal cycling is repeated multiple times for a given sample to achieve different undercooling levels and gather reproducibility statistics. Alternatively, the sample with a particular thermal history can be saved for the microstructure analysis.

A qualitative description of the phase transformations discussed in the previous paragraph demands a critical examination of the temperature and diffraction patterns before conducting any quantitative analysis. The sample temperature was measured at an emissivity of 0.2 in single-color mode. The pyrometer data were calibrated against a known reference temperature using the equation provided in the supporting information, as described in the following. The reproducibility of the reference temperature from cycle to cycle limits the accuracy of the temperature measurements to approximately ±10 K. For elemental Fe, the *T*_γ–δ_ temperature, observed during heating and cooling, is used to obtain accurate temperature values in the solid state. The temperature of the liquid phase is calibrated using *T*_m_ measured just before complete melting. Extrapolation of calibration at *T*_m_ into the solid region (gray line in Fig. 6 ▸) underestimates the *T*_γ–δ_ temperature by ∼25 K (2%). The difference in spectral emissivities between the liquid and solid phases, along with the inward-directed temperature gradients that occur during residual solidification (with the surface temperature being lower than *T*_m_), limits the accuracy of temperature measurements in the semi-solid regions, *i.e.* during melting and solidification. Changes in the surface roughness during solid–liquid transformation can further complicate temperature readings (see melting plateau in Fig. 6 ▸). Furthermore, the measured temperature can be impacted (apparently increased) in the presence of residual oxides on the surface of the liquid sample, as they usually exhibit higher emissivity than metals (not present in current samples). Finally, the movements of the levitating sample can lead to oscillations in the measured temperature [post-recalescence part in Fig. 9(*c*)]. Emissivity variations between different phases and surface roughness are minimized in two-color pyrometer mode. However, this mode also reduces the accessible temperature range for a given pyrometer and lowers accuracy at lower temperatures. More importantly, the assumption of a constant emissivity ratio does not hold for most materials, limiting its benefits for temperature measurements in levitation experiments.

**Figure 7:**
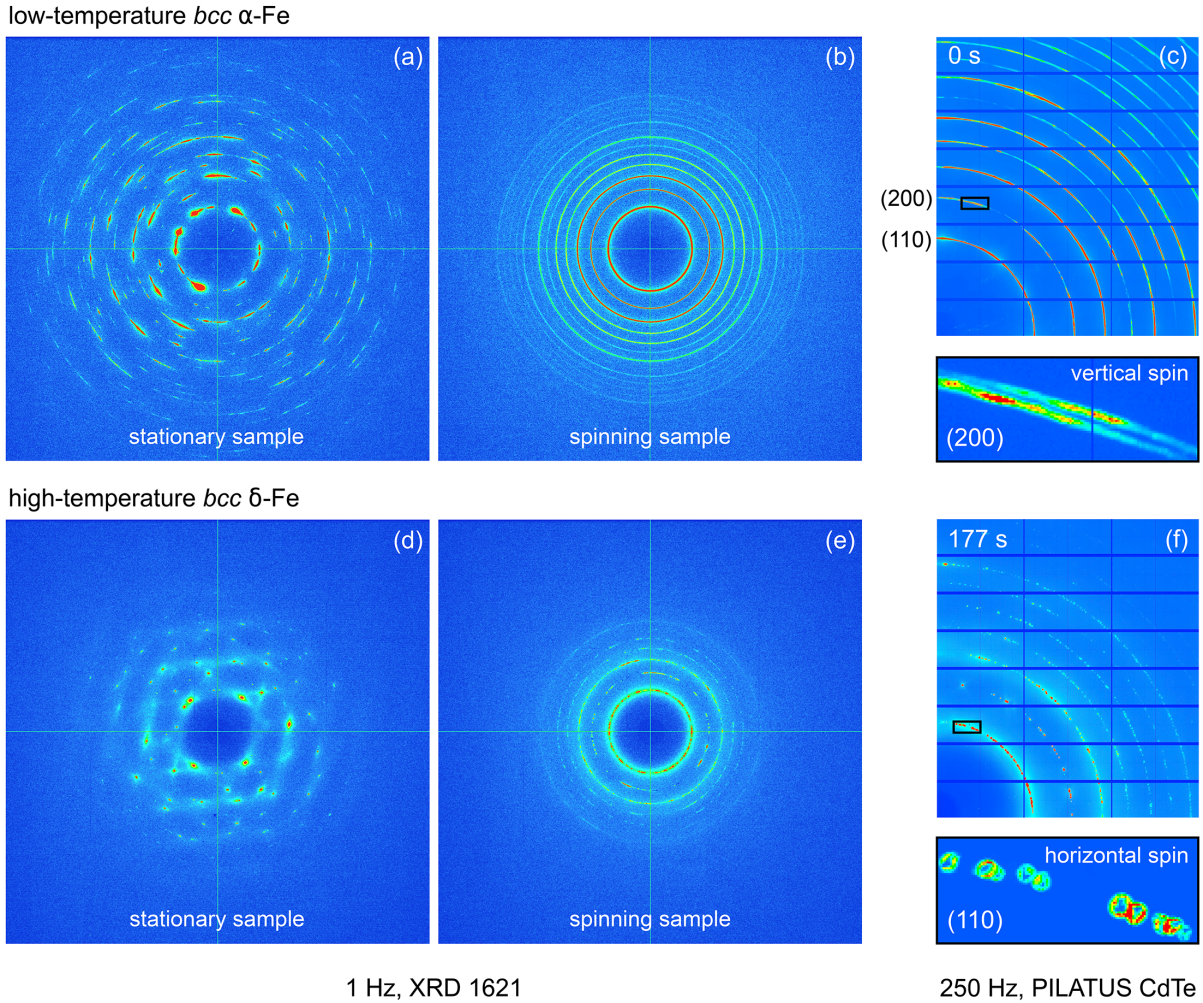
Two-dimensional diffraction patterns of the b.c.c.-Fe taken during heating of the α phase (*a*–*c*) and solidification of the δ phase (*b*–*f*) on stationary and spinning samples. The off-center geometry captured by the PILATUS detector during the cycle shown in Fig. 6 ▸ is compared with the symmetrical pattern acquired with the XRD 1621 detector during a similar thermal cycle. The sensitive areas of both detectors are displayed to scale. A magnified view of the areas indicated by the black boxes is shown in the corresponding bottom panels.

Selected two-dimensional diffraction patterns of the low- and high-temperature b.c.c. structure of pure Fe are shown in Fig. 7 ▸. The off-center scattering axis geometry captured by the PILATUS detector during the cycle in Fig. 6 ▸ is compared with the symmetrically centered patterns obtained using the XRD 1621 detector in a similar thermal cycle (see Section 3.5 for detector comparison). Fig. 7 ▸(*a*) demonstrates typical pattern characteristic of the polycrystalline microstructure of room temperature α. In contrast, the structure shown in Fig. 7 ▸(*d*) indicates that the δ phase forms an intermediate γ-Fe as a single-crystal. The details of the transformations between the two microstructure types become obscured as the levitating sample develops spin around the vertical axis due to imperfections in the symmetry of the magnetic field generated by the coil. Furthermore, density redistribution during melting and solidification, along with the associated shift of the center of mass, also leads to the rotational motion of a freely suspended sample, regardless of levitation type. In the liquid state, particularly during deep undercooling, viscous dissipation and energy loss from surface oscillations contribute to the damping of sample rotation. Under favorable flow conditions, the drag from the cooling gas can further slow the rotation but cannot fully control it. Ultimately, sample rotation is advantageous for the quantitative analysis of the measured intensity, averaging the single- or polycrystalline structure to the powder diffraction-like rings as shown Figs. 7 ▸(*b*) and 7 ▸(*e*) (compare patterns from a stationary and spinning sample) and effectively increasing the gauge volume probed by the beam during individual measurements as illustrated schematically in Fig. 5 ▸. At the same time, a stationary sample or one spinning slower than the detector’s acquisition rate provides more information on the sample microstructure and its evolution, *e.g.* crystal dissociation, grain refinement, and crystallographic phase relation.

**Figure 8:**
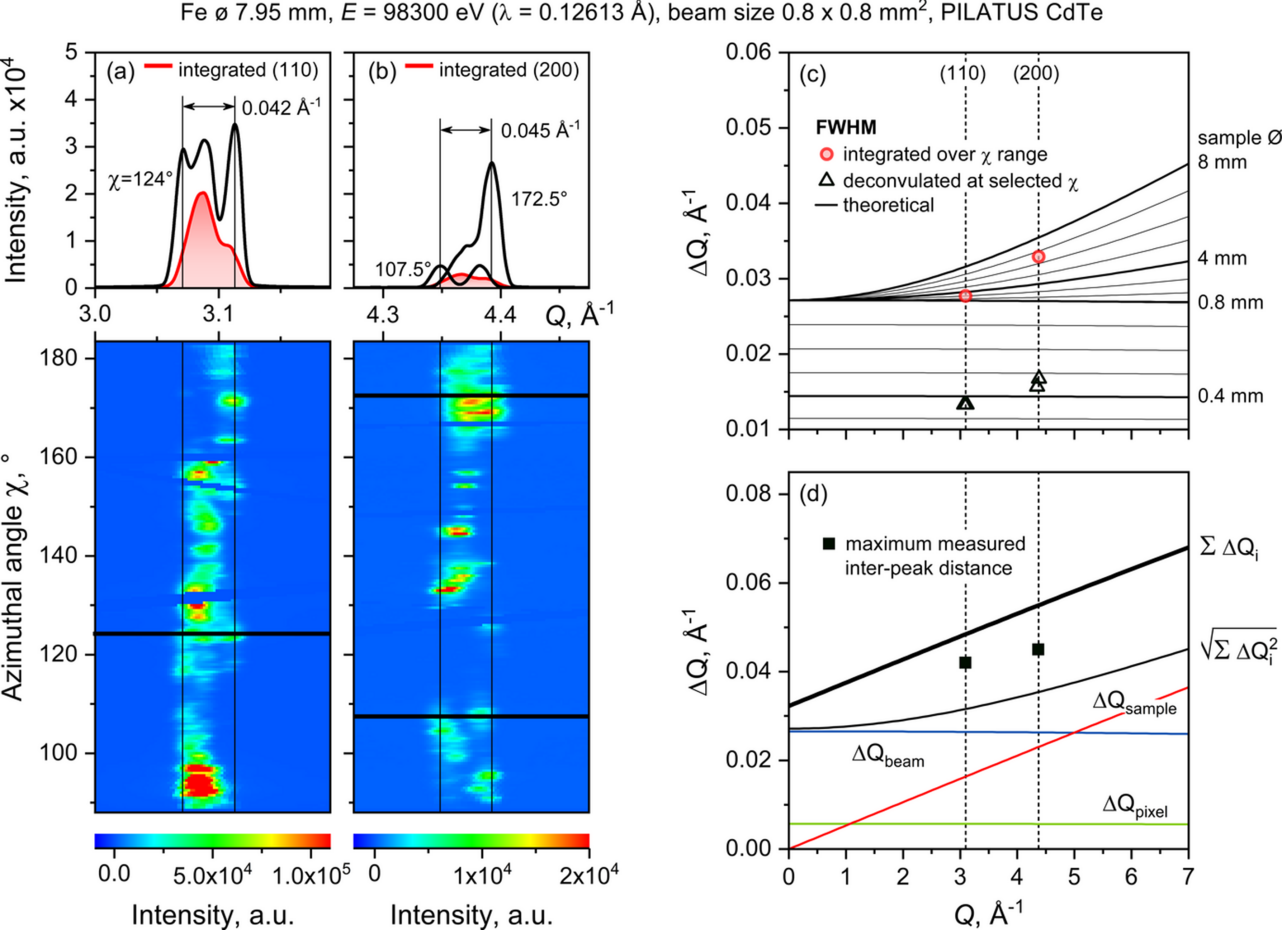
Intensity distributions along the (110) (*a*) and (200) (*b*) rings of an α-Fe pattern from Fig. 7 ▸(*c*) as a function of azimuthal angle χ. Upper panels compare profiles integrated over the full χ range (red) with profiles at selected azimuthal angles (black); the asymmetry of the integrated profile reflects limited statistics due to a reduced χ range measured in the current geometry. FWHM values from deconvoluted profiles at selected χ angles are compared with calculated resolution functions for different effective sample sizes (*c*). Maximum inter-peak distance measured across the χ range compared with the sum of the main broadening contributions (*d*).

In this context, it is essential to distinguish between the structural features and the artifacts arising from the sample, beam and pixel sizes, which are most clearly observed at higher spatial resolution, as shown in Figs. 7 ▸(*c*) and 7 ▸(*f*). Depending on their position within the gauge volume (Fig. 5 ▸), the diffracted intensity from the grains of similar orientation will strike the detector at varying distances from the beam center. Such differences in effective grain-to-detector distance, exacerbated by the pixel size and oblique incidence, combine to cause peak broadening in fully polycrystalline samples such as LaB_6_. With fewer variations of grain orientations present in a stationary or slowly rotating sample, the distribution of the diffraction spots along the ring will be less homogeneous and can lead to an apparent splitting of the diffraction ring, as shown in the inset of Fig. 7 ▸(*c*). The corresponding azimuthal intensity distributions along the (110) and (200) rings of α-Fe are shown in Figs. 8 ▸(*a*) and 8 ▸(*b*), respectively. Multiple peaks contributing to the overall intensity profiles are illustrated for the selected χ angles. The widths of the deconvoluted peaks map to sample sizes of a few hundred micrometres, as estimated from the theoretical FWHM values calculated for the given experimental geometry and varying sample sizes [Fig. 8 ▸(*c*)]. Taking into account additional contributions to the broadening from internal strain and structural disorder, these peaks can be attributed to individual grains. Note that the maximum differences in the positions of individual peaks along the ring do not exceed the sum of the individual contributions used to calculate the theoretical peak broadening [Fig. 8 ▸(*d*)]. The asymmetry of the intensity profiles, integrated over the entire measured χ range, illustrates the drawbacks of reduced azimuthal coverage and hampers an adequate fit with a single analytical profile. These limitations should always be considered in both qualitative and quantitative analyses of diffracted intensities.

Vertical elongation of the spinning liquid sample can lead to a sudden shift in the center of mass, altering the sample’s moment of inertia and flipping it into a horizontal plane. When the horizontal rotation axis is nearly aligned with the scattering axis, the diffraction spot from a specific grain will trace a ring-like shape on the detector [Fig. 7 ▸(*f*)], provided that the spinning rate exceeds the pattern acquisition rate. This only affects grains that fall within the gauge volume defined by the size of the beam. Caused by the effects arising from the final beam, sample size, and rotation of the sample in a plane parallel to that of the detector, this artifact does not provide meaningful intensity measurements and must be excluded from further quantitative analysis.

High-time resolution diffraction measurements are aimed to capture rapid transformations associated with the corresponding thermal events, *e.g.* recalescence during rapid solidification. Further reduction of the data acquired at 250–500 Hz to 5–10 Hz averages the intensity of diffraction patterns for subsequent quantitative analysis.

### 3.2. Fe–Co

**Figure 9:**
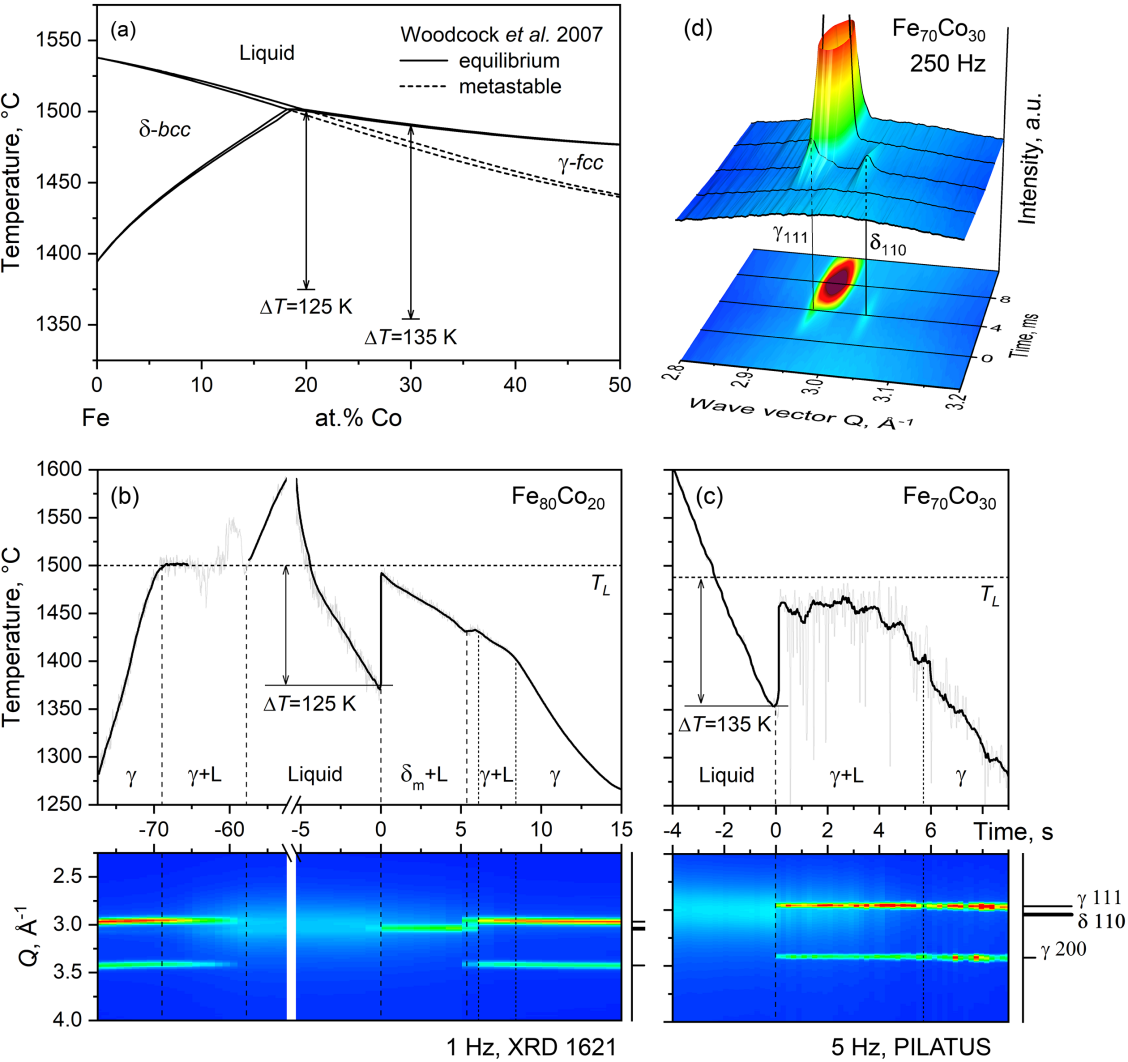
Metastable phase formation in undercooled Fe–Co alloys. (*a*) Part of the equilibrium Fe–Co phase diagram with metastable extension of the δ phase solidus and liquidus lines. Arrows indicate melt undercooling achieved during diffraction EML experiments in the Fe_80_Co_20_ (*b*) and Fe_70_Co_30_ (*c*) alloys. Temperature is synchronized in time with the corresponding evolution of the sample structure, depicted as the intensity contour plots of selected reflections. Formation of the b.c.c. structure during solidification of the Fe_80_Co_20_ alloy (*b*), not observed upon melting, confirms the metastable nature of the δ_m_ phase. In the course of solidification of the Fe_70_Co_30_ alloy the δ_m_ phase is not apparent at reduced time resolution used in (*c*). However, its formation during recalescence is captured in a spectrum acquired at 250 Hz, represented as a 3D contour plot of the main reflections in (*d*).

The phase formation in pure Fe, discussed above, changes with the addition of Co. Following the binary Fe–Co phase diagram, the high-temperature δ-b.c.c. phase remains stable only up to 18 at.% Co [Fig. 9 ▸(*a*)]. From concentrations of ∼19.6 at.% Co, the γ-f.c.c. phase forms a stable equilibrium with the liquid. Accordingly, during heating of the Fe_80_Co_20_ alloy, γ phase reflections are observed up until complete melting [Fig. 9 ▸(*b*)]. However, upon the following solidification, the primary phase formed from an undercooled liquid exhibits a b.c.c. structure inferring the formation of the metastable δ_m_ phase. It takes more than 5 s for the equilibrium γ phase to nucleate and take over the δ_m_. Formation of the δ_m_ phase at temperatures below the metastable extension of the corresponding liquidus line is thermodynamically feasible (Woodcock *et al.*, 2007 ▸). Furthermore, the lower interfacial energy associated with the nucleation of a more open b.c.c. structure, compared with close-packed f.c.c., promotes faster kinetics of its formation. At the same time, with increasing Co content, the difference between the liquidus lines of the stable and metastable phases becomes larger, which accelerates the driving force for the δ_m_-to-γ phase transformation. This leads to an exponential decrease in the lifetime of the metastable phase (Kreischer & Volkmann, 2021 ▸). Thus, at an acquisition rate of 5 Hz, only γ phase reflections are detectable during the solidification of the Fe_70_Co_30_ alloy, despite the undercooling being similar to that of Fe_80_Co_20_ [compare Figs. 9 ▸(*b*) and 9 ▸(*c*)]. The b.c.c. reflections are first distinguished as the acquisition rate increases to 250 Hz. As shown in Fig. 9 ▸(*d*), the diffraction pattern collected within 4 ms at the onset of recalescence in Fe_70_Co_30_ includes reflections from both the δ_m_ and γ phases. In the subsequent spectrum, only γ reflections are present, indicating that the transformation is completed within a few milliseconds.

The metastable δ_m_ phase was first detected in rapidly solidified Fe–Co submicrometre droplets over three decades ago (Kim & Kelly, 1991 ▸). Further studies have indicated that, although it completely transforms into the stable γ phase at lower cooling rates, its formation impacts the final microstructure, thereby influencing material performance in applications. For industrially relevant magnetic Fe–Co alloys, the thermodynamics and kinetics of the metastable δ_m_ phase have been primarily studied using levitation methods, which provide unique access to deeply undercooled bulk samples suitable for *in situ* diagnostics (Kreischer & Volkmann, 2021 ▸). High-speed video imaging at kHz frame rates reveals metastable phase solidification through thermal contrast with the stable phase, but lacks structural information. Time-resolved diffraction studies on electromagnetically levitated Fe–Co alloys reported here offer the first unambiguous observation of the structure of the metastable δ_m_ phase formed in this system. The high temporal resolution of the PILATUS detector was essential for detecting the metastable phase at high Co concentrations, where its lifetime is reduced to only a few milliseconds. Due to the stochastic nature of spontaneous nucleation, the metastable phase was captured only in a subset of the four cycles performed for each sample, as shown in Fig. 9 ▸. These results were confirmed in subsequent studies, which will be reported elsewhere.

### 3.3. Other systems

Further capabilities of the mobile EML diffraction setup are detailed in the following publications with emphasis on liquid thermophysical properties (Jeon *et al.*, 2020 ▸), metastable phase formation (Matson *et al.*, 2017 ▸; Jeon *et al.*, 2019 ▸; Baker *et al.*, 2021 ▸), and phase transformation in complex alloys (Mattern *et al.*, 2018 ▸; Andreoli *et al.*, 2021*b* ▸; Andreoli *et al.*, 2021*a* ▸; Wolff-Goodrich *et al.*, 2022 ▸).

### 3.4. Challenges of liquid structure measurement on levitating droplet

The structural information of the liquid sample is conveyed in the diffuse diffracted intensity collected across the entire range of measured reciprocal space. Experiment- and material-specific intensity corrections followed by a normalization procedure yield the liquid structure factor, *S*(*Q*). The real-space distribution of interatomic distances, the pair distribution function (PDF), is obtained from measured *S*(*Q*) via Fourier transformation. Thus, the quality of the PDF is determined by both the maximum range of the measured momentum transfer, *Q*_max_, and the accuracy of the intensity corrections and normalization procedure applied (Hoyer *et al.*, 2001 ▸).

The inverse relationship with the wavevector allows the short wavelengths of high-energy X-rays used in levitation experiments to encompass broad reciprocal space. This coverage can be further maximized by reducing the sample-to-detector distance and off-centering the detector relative to the beam.

On the detector side, the flat-field response is a particularly critical correction for the weak diffuse intensity signal of disordered structures. To account for potential changes in detector gain during solidification, which may occur with rapid alterations of strong structural features (Bragg reflections), dedicated flat-field measurements, as discussed in Section 3.5, should be performed before each experimental cycle aimed at liquid structure analysis.

Furthermore, to obtain an accurate PDF, all effects of the scattered radiation’s oblique incidence on a flat detector of a given thickness must be considered. In addition to standard solid angle and *Q*-dependent intensity corrections, corrections of the *Q* values to account for the increased spreading of the detected scattering angle are required (see Section 3.5).

The background scattering from the sample environment, *i.e.* air, levitation chamber, *etc*., is routinely accounted for by subtracting the detector image acquired with an empty chamber under the same experimental conditions. It is important to note that, when working with a strongly absorbing sample, this method may lead to an overestimation of the actual background (Skinner *et al.*, 2012 ▸).

Intensity corrections specific to levitation experiments must consider the asymmetric transmission geometry resulting from the limitations of sample position control. As demonstrated by Bendert *et al.* (Bendert *et al.*, 2013*a* ▸; Bendert *et al.*, 2013*b* ▸), for isotropically scattering disordered materials, the measured asymmetry of the diffracted intensity can be used to deduce sample self-absorption and secondary (multiple) scattering for a particular geometric configuration. Prior to the analysis, the two-dimensional data should be corrected for all potential sources of intensity gradients. This includes geometric corrections and corrections for the X-ray beam polarization, which are typically performed during the integration step by software used for data reduction.

The peculiarities of intensity corrections for Compton scattering and fluorescence measured with area detectors are detailed by Skinner *et al.* (2012 ▸). Note that for energy-discriminating detectors, such as the PILATUS, the fluorescence background, which arises from the re-emission of absorbed incident photons at lower energies, can be eliminated by setting a material-appropriate threshold.

The aforementioned corrections to the experimental diffraction data from the levitating liquid sample are essential for the normalization procedure to obtain a reliable structure factor and, consequently, an accurate pair distribution function on an absolute scale.

### 3.5. Detector-specific considerations for structural studies on levitating samples

The following section compares two flat area detector types and the associated correction procedures, in the context of time-resolved diffraction experiments on levitated samples undergoing dynamic structural changes.

The XRD 1621 represents a class of indirect detectors that use thallium-doped caesium iodide as a scintillator material to convert high-energy X-rays into visible light prior to detection by an array of amorphous silicon (a-Si) photodiodes. Deposited directly onto the photodiodes coupled with thin-film transistors (TFTs), the needle-like structure of the CsI(Tl) crystals decreases the lateral divergence of the incident X-rays and the re-emitted fluorescence photons while maximizing the photoabsorption and quantum efficiency of a-Si.

Despite their overall strong performance, indirect detectors are less efficient at counting incident photons than more recently developed direct X-ray detectors, such as the PILATUS CdTe. To ensure good quantum efficiency for energies up to 100 keV, the detector uses 1000 µm thick cadmium telluride (CdTe) single crystals as the sensor material. An electric field captures the charge generated by an individual photon within a 172 µm sensor pixel, minimizing the loss and spread of the signal. The PILATUS detector utilizes hybrid photon counting technology with CMOS electronics, eliminating dark current and readout noise. It provides nearly non-paralyzable photon counting across a dynamic range of 20 bits at frame rates of up to 500 Hz. All these parameters substantially surpass those of the XRD 1621 detector given in Table 1 ▸. Moreover, the residual readout noise of the TFT-based electronics increases the signal-to-noise ratio of the indirect detector, while the inherent dark current requires offset corrections through the dark image subtraction. In contrast, PILATUS delivers sharp images free of artifacts and at higher spatial and temporal resolution, with an adjustable low-energy threshold. These characteristics are essential for accurately determining reflections from crystalline phases, their evolution, and discrimination during dynamic events, such as rapid phase transformations.

On the other hand, the monolithic flat panel of the a-Si detector features a sensitive area of 409.6 mm × 409.6 mm, which is more than double the area of 253.7 mm × 288.8 mm covered by the PILATUS (see to-scale comparison in Fig. 7 ▸). Another drawback of the direct photon counting detector is its modular structure. Composed of individual modules, each containing two 42 mm × 34 mm CdTe single crystals with a 3-pixel gap in between, the modules themselves are separated by significantly larger gaps of 7 and 17 pixels in the horizontal and vertical directions, respectively. The network of inactive regions takes up 8.5% of the total detector area. These blind regions can hinder the detection of the crystalline phase represented by a few spots (*e.g.* single crystal). By compromising on time resolution, the larger area of the XRD 1621 detector, with no significant inactive regions, can be advantageous for structural studies of non-crystalline states, such as liquids and glasses. This capability allows for capturing a larger reciprocal space with improved counting statistics.

In scattering experiments with flat area detectors, the increased travel distance and oblique incidence of photons at higher diffraction angles reduce the solid angle and the corresponding flux captured by each pixel. Corresponding intensity corrections are typically applied at the integration stage by data reduction software (*e.g.* solid angle correction to intensities in *pyFAI*).

Furthermore, oblique incidence on the detector’s active layer results in effects that increase detection efficiency due to the longer attenuation path of photons while also decreasing spatial resolution because of the signal spreading within the detector plane. To ensure accurate data analysis, angle-dependent intensity corrections, known as detector attenuation or obliqueness corrections (Skinner *et al.*, 2012 ▸; Bendert *et al.*, 2013*b* ▸), should be complemented by corresponding corrections of the diffraction angle (Marlton *et al.*, 2019 ▸). The effects will be more pronounced in the PILATUS detector, as it has an active layer twice as thick as that of the XRD 1621 detector.

Accurate intensity measurements using either type of area detector must account for the non-uniformity of the X-ray beam and the inconsistent response of individual pixels. Originating from variations in gain, losses, or trapping of charge, and fluctuations in the performance of the readout electronics, differences in pixel sensitivities are further amplified by prolonged exposure to high-energy radiation. Standard flat-field measurements used to correct these effects require uniform illumination across the entire detector area. Available sources of homogeneous radiation usually provide flat-field corrections for low-energy photon experiments, which must be interpolated for higher-energy ranges (Skinner *et al.*, 2012 ▸). Alternatively, a source that produces a non-flat yet isotropic structure can be used at any arbitrary X-ray energy. Weng *et al.* (2023 ▸) have proposed a methodology for determining the flat-field correction using radially symmetric diffraction patterns from an amorphous scatterer (*e.g.* silicate glass). While this approach relies on the linearity of the detector’s response to varying intensities, it offers a practical solution for addressing the changes in pixel performance over time, which is particularly vital for high-energy experiments.

Continuous dynamic experiments that involve acquiring successive patterns over extended periods pose further challenges for detector performance. In detectors that utilize scintillators, delays in light emission, known as afterglow, can cause residual charge from one measurement to bleed into subsequent ones. Moreover, both types of detectors suffer from radiation damage when subjected to photon fluxes that exceed their respective count levels. Permanently damaged pixels are excluded from analysis through masking techniques implemented in *pyFAI* or similar software. The value of a defective pixel can be substituted with the average or a weighted interpolation of neighboring pixels, enhancing the overall accuracy of the measured intensity. Non-permanently damaged pixels change their response over time, *e.g.* due to the release of trapped excited states. This process can be either accelerated or mitigated following the procedures suggested by Skinner *et al.* (2012 ▸).

Due to the dynamic nature of the levitation experiments, characterized by dramatic structural changes (*e.g.* spontaneous nucleation and rapid growth of the crystalline phase from the liquid), oversaturation in specific detector areas is difficult to avoid. To reduce this effect and avoid pixel damage, the use of a proper attenuator in the incident beam is needed.

## 4. Summary

The mobile electromagnetic levitator presented for time-resolved high-energy X-ray diffraction studies using flat area detectors offers a versatile tool for *in situ* observation of structure formation across a wide range of materials. Its compact and multipurpose design allows for easy transport, quick assembly, and adjustment at the synchrotron beamline. Batch processing of multiple samples streamlines experiments and optimizes the use of allocated operational time. The mobile EML has been successfully deployed at beamline P07 at PETRA III (DESY), which delivered a high-energy monochromatic X-ray beam of sufficient flux to implement transmission scattering geometry. A comparative analysis of the indirect and direct flat area detectors currently available for dynamic diffraction studies is provided with particular consideration of their application in levitation experiments. The analytical description of measured spatial resolution offers a practical guide for the experimental design. The intensity corrections and additional measurements required to obtain high-quality total structure factors from scattering experiments on levitating liquid droplets are discussed. Key artifacts characteristic of Bragg diffraction patterns during levitation processing are surveyed based on experiments conducted with pure Fe. Finally, the formation of the metastable b.c.c. phase in undercooled Fe–Co alloys, with a lifetime of a few milliseconds, has been directly observed for the first time. This has demonstrated the capabilities of the mobile EML diffraction setup equipped with a rapid area detector to capture dynamic structural changes occurring during non-equilibrium solidification.

## Supplementary Material

Supporting information. DOI: 10.1107/S1600577526003541/ye5078sup1.pdf

Python code to calculate the total instrumental resolution and its contributions. DOI: 10.1107/S1600577526003541/ye5078sup2.zip

## Figures and Tables

**Table 1:** Comparative characteristics of the area detectors used for time-resolved XRD on levitated samples

	PerkinElmer XRD 1621	PILATUS3 X CdTe 2M
Sensitive area	409.6 mm × 409.6 mm	253.7 mm × 288.8 mm
Inactive area	2.3%	8.5%
Pixel number	2048 × 2048	1475 × 1670
Pixel size	200 µm	172 µm
Dynamic range	16 bit	20 bit
Point-spread function	1.1 pixel	1 pixel
Maximum frame rate	15 Hz (30 Hz†)	250 Hz (500 Hz‡)

† At 400 µm effective pixel size via 2 × 2 binning.

‡ For image area reduced to 8 of the 24 modules.

## Data Availability

Relevant data are included in the supporting information submitted with this publication.
