# Supplementary material for: Mobile electromagnetic levitator for time-resolved in situ X-ray diffraction studies of high-temperature phase transformations
Source: J Synchrotron Radiat. 2026 May 6;33(Pt 4):1022–35. doi: 10.1107/S1600577526003541 (PMC13344540; doi:10.1107/S1600577526003541)
Supplement: Supplementary file 1 [file s-33-01022-sup1.pdf]

JOURNAL OF SYNCHROTRON RADIATION

Volume 33 (2026)

Supporting information for article:

Mobile electromagnetic levitator for time-resolved *in situ* X-ray diffraction studies of high-temperature phase transformations

Olga Shuleshova, Ivan Kaban, Steffen Ziller, Uwe Reinhold, Ann-Christin Dippel, Olof Gutowski and Martin von Zimmermann

Mobile electromagnetic levitator optimized for time-resolved *in situ* X-ray diffraction studies of high-temperature phase transformations

Olga Shuleshova[a], Ivan Kaban[a], Steffen Ziller[a], Uwe Reinhold[a], Ann-Christin Dippel[b], Olof Gutowski[b], and Martin von Zimmermann[b]

[a]Leibniz Institute for Solid State and Materials Research Dresden, Helmholtzstr. 20, 01069 Dresden, Germany
[b]Deutsches Elektronen-Synchrotron DESY, Notkestr. 85, 22603 Hamburg, Germany

1 Mobile EML

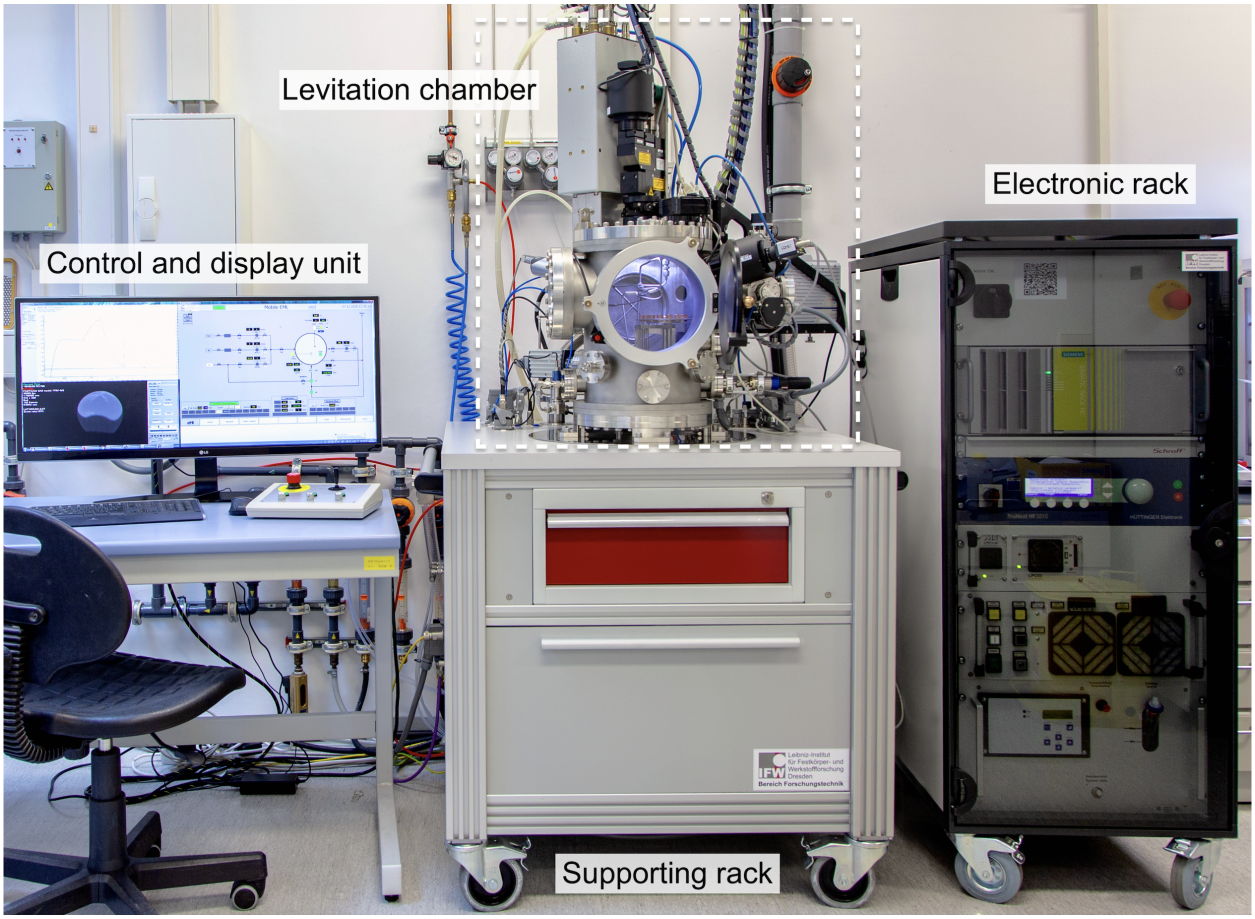

Figure 1: Mobile electromagnetic levitation facility at IFW Dresden

2 Temperature calibration

Temperature measurements with an infrared pyrometer operating at a wavelength λ_{pyro} require knowledge of the corresponding spectral emissivity of the material. Deviations between the assumed emissivity $\varepsilon_{\text{used}}$ and the actual emissivity $\varepsilon_{\text{true}}$ lead to systematic errors in the measured temperature. These can be corrected using the relation:

$$T_{\text{corrected}} = \left[\frac{1}{T_{\text{measured}}} + \frac{\lambda_{\text{pyro}}}{C_2} \ln\left(\frac{\varepsilon_{\text{true}}}{\varepsilon_{\text{used}}}\right)\right]^{-1},$$

where $C_2 = 1.438 \times 10^4\,\mu\text{m} \cdot \text{K}$ is Planck's second radiation constant.

An alternative calibration approach uses a known reference temperature that can be clearly identified during the experiment, such as the liquidus point. The corrected temperature is calculated as:

$$T_{\text{corrected}} = \left[\frac{1}{T_{\text{measured}}} + \left(\frac{1}{T^{\text{ref}}_{\text{true}}} - \frac{1}{T^{\text{ref}}_{\text{measured}}}\right)\right]^{-1},$$

where $T^{\text{ref}}_{\text{measured}}$ and $T^{\text{ref}}_{\text{true}}$ denote the measured and actual temperatures at the reference point, respectively.

3 Calculation of the instrumental resolution function

The instrumental resolution function of the diffraction levitation setup with an area detector is modeled as a Gaussian convolution of independent broadening contributions to the total full width at half-maximum (FWHM) of a hkl reflection:

$$\Delta 2\theta_{hkl}^2 = \Delta 2\theta_{\text{sample}}^2 + \Delta 2\theta_{\text{beam}}^2 + \Delta 2\theta_{\text{pixel}}^2 + \delta^2 + w_{hkl}^2 \,. \tag{1}$$

Assuming negligible contributions from beam divergence δ and the intrinsic width of the LaB_6 reflections w_{hkl}, the total broadening at a fixed sample-to-detector distance (SDD) is attributed to the sample diameter s, beam size b[1], and detector pixel size p (including the point spread function). These contributions are derived from the experimental geometry, depicted in Fig. 5 of the main article, and expressed as functions of the scattering angle 2θ[2]:

$$\Delta 2\theta_{\text{sample}} = \arctan\left(\tan 2\theta + \frac{\text{s} \cdot \tan 2\theta}{SDD}\right) - 2\theta, \tag{2}$$

$$\Delta 2\theta_{\text{beam}} = \arctan\left(\tan 2\theta + \frac{\text{b}}{SDD}\right) - 2\theta, \tag{3}$$

$$\Delta 2\theta_{\text{pixel}} = \arctan\left(\tan 2\theta + \frac{\text{p}}{SDD}\right) - 2\theta. \tag{4}$$

Python code to calculate the total instrumental resolution and its contributions is provided in a separate file `Instrumental_resolution.py`.

[1]When Bragg conditions are satisfied only for individual grains within the sample, the effective beam size is reduced to that of the grain size.

[2]The spherical sample is treated as a flat plate of thickness equal to its diameter, and the beam footprint is considered circular. Although these approximations have little effect on the FWHM, the azimuthal variation of the beam width reaching $\sqrt{2}$ of the beam size along the diagonal must be accounted for when analysing reflection positions along the Debye–Scherrer ring.

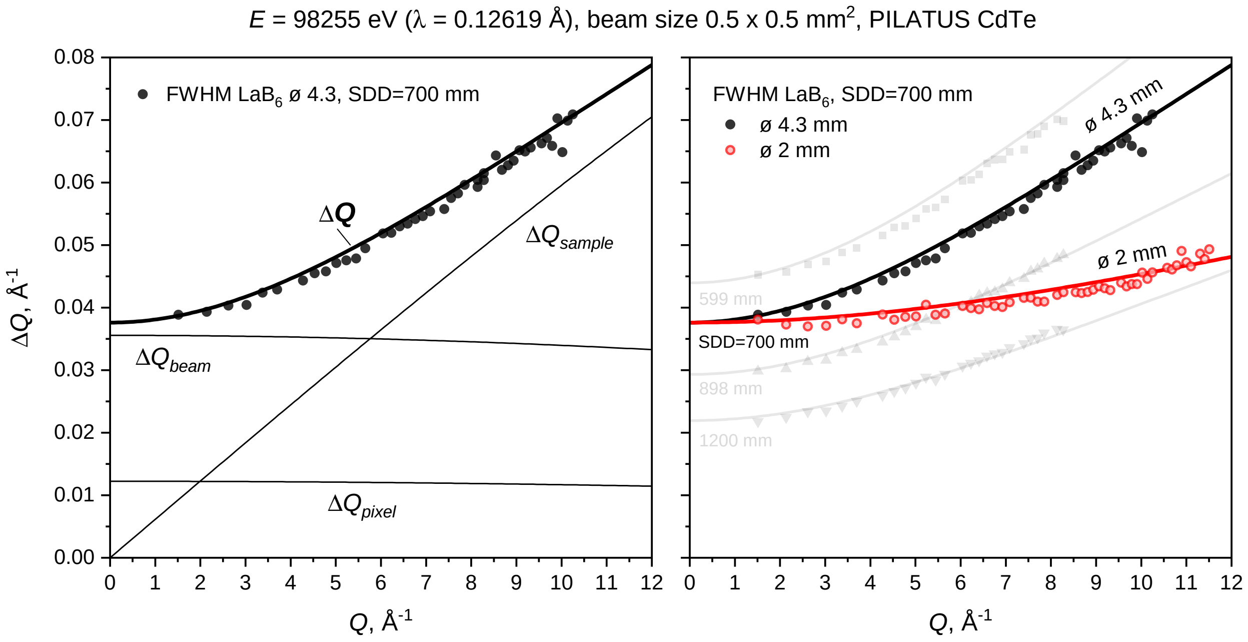

Figure 2: (Left panel) Resolution function of the detector calculated using Eq. (1) as a Gaussian convolution of individual broadening contributions from Eqs. (2)–(4), with 2θ converted to the wave vector $Q = 4\pi \sin\theta/\lambda$; compared to FWHM measurements from a standard. (Right panel) Effect of sample diameter on the overall resolution of the diffraction setup.
